# Effect of Ochratoxin A on Body Weight, Feed Intake and Feed Conversion in Broiler Chicken

**DOI:** 10.4061/2010/590432

**Published:** 2010-12-27

**Authors:** Sigamani Masilamani Sakthivelan, Ganne Venkata Sudhakar Rao

**Affiliations:** ^1^Department of Pathology, Veterinary College and Research Institute, Tamil Nadu Veterinary and Animal Sciences University, Tamil Nadu, Namakkal 637 002, India; ^2^Department of Pathology, Madras Veterinary College, Vepery, Chennai 600 007, India

## Abstract

The effect of ochratoxin A (OA) on the body weight, feed intake, and feed conversion was investigated in broiler chicken fed dietary levels of OA at 0, 1, and 2 ppm for 28 days from hatch. Feeding OA significantly reduced the growth rate of broiler chicken. The reduction was observed from the first week onwards in OA-treated groups. Feed consumption and feed conversion also showed a diminishing trend from the first week of feeding toxin. Its implication on the performance of broiler chicken is discussed.

## 1. Introduction

Ochratoxin A (OA), a nephrotoxic mycotoxin mainly produced by *Aspergillus ochraceus and Penicillium viridicatum, *has been shown to contaminate a wide variety of cereals and feed stuffs and is extremely toxic to domestic fowls [1] and swine [2]. Spontaneous occurrence of OA in feed and feed stuffs have been reported and OA has been implicated in field outbreaks of mycotoxicosis resulting in poor growth rate and poor feed efficiency [3]. The effects of OA in poultry were found to be quite pronounced in young broiler chicks [4].

An attempt is, therefore, made to study the nature of effect of experimental OA toxicity in young broiler chicks. This investigation seeks to study the pattern of change elicited by OA in diet on feed intake, feed conversion, and body weight in broiler chicken.

## 2. Material and Methods

Sixty-day-old broiler chicks (Vencob, India) received from a commercial hatchery were randomly divided into six replicates of ten chicks each and housed in well-ventilated clean cages with optimum, continuous lighting. The chicks were provided with feed and water, *ad libitum.*

### 2.1. Standard Toxin

Pure ochratoxin A obtained from M/s. Sigma chemicals, USA was used as the standard for the experiment.

### 2.2. Fungal Culture

*Aspergillus ochraceus* NRRL 3174 obtained from S. W. Peterson, Microbiologist, Microbial properties research, United States Department of Agriculture, Agricultural Research Service, 1815, North University Street, Peoria, Illinois 61604, USA, in lyophilized form was used as the seed culture.

The fungus was maintained by subculturing at 10 days interval in Czapek Dox agar supplemented with 20 per cent sucrose and 0.7 per cent yeast culture powder, as slant culture [5]. 

### 2.3. Production of Ochratoxin A

Ochratoxin A was produced on broken wheat [6].

One hundred g of the substrate( broken wheat) was taken in 500 ml conical flasks and soaked in 80 ml tap water for 2 hours. The flasks were autoclaved at 15 psi for 15 min and cooled. Five ml of distilled water was added to slant cultures of the fungi which was already prepared, and one ml of this was inoculated into the conical flasks. The flasks were kept at room temperature in slanting position, and hands taken vigorously six to ten times a day to avoid clump formation. On the fourteenth day, the mouldy substrate was briefly steamed for five minutes to kill the spores and dried overnight at 60°C in a hot air oven. The mouldy substrate was powdered and the ochratoxin content was estimated by the method of Manning and Wyatt [7].

### 2.4. Estimation of Toxin

Quantity of ochratoxin A in mouldy substrate was estimated by the method followed by Manning and Wyatt [7]. Ten grams of mouldy feed was blended with 150 ml of chloroform methanol mixture (1 : 1) for 10 minutes and filtered. The filtrate was extracted twice using 75 ml of 0.5 M sodium bicarbonate in a separating funnel. The sodium bicarbonate fractions were pooled and acidified to pH 1.5 with concentrated HCl and extracted twice with 75 ml chloroform. The chloroform extracts were combined, orated to dryness, and dissolved in 2 ml benzene. An aliquot of the benzene solution and an ochratoxin A standard solution were serially diluted and spotted on a precoated silica gel 60 thin layer chromatography plates (0.2 mm thickness). The plates were developed in benzene-acetic acid (9 : 1), allowed to dry and examined under longwave ultraviolet light. The OA content was quantified by both spot comparison and dilution to extinction methods.

The experimental design consisted of three dietary levels of OA at 0, 1 and 2 ppm for 28 days from hatch. OA produced in broken wheat [6]. Known amount of powdered wheat culture containing OA were incorporated into OA free broiler starter mash to yield 1 ppm and 2 ppm OA. One control diet was also prepared. The diet contained 23 per cent crude protein.

 Feed consumption, feed conversion, and body weight were worked out for each group. Blood samples collected by cardiac puncture were allowed to clot and centrifuged for 20 minutes at 1500 rpm to separate the sera. Pooled serum samples were used to determine the total serum protein and albumin [8].

### 2.5. Statistical Analysis

The data generated from different parameters of the experimental study were subjected to one way analysis of variance(ANOVA) by using SPSS software.

## 3. Result and Discussion

### 3.1. Body Weight

Feeding OA significantly reduced the growth rate of broiler chickens (Table 1). The reduction was observed from the first week onwards in OA treated groups. However, reduction observed between the control and 1 ppm level at first week was insignificant.

Similar observations of growth depression with varying levels of dietary ochratoxin A ranging from 0.8 to 10 *μ*g per gram were also studied by Huff et al. [9], Prior et al. [10], Dwivedi and Burns [11], Kubena et al. [12], Manning and Wyatt [7], and Huff et al. [4].

The reduced weight gain induced by OA could be attributed to the reduced feed intake as observed in this study. Impaired protein metabolism was implicated as the main cause [13]. It was observed that OA competes with phenylalanine for binding sites on the Phenylalanyl transfer-RNA-synthetase enzyme, thus inhibiting protein synthesis [4].

### 3.2. Feed Consumption

Feeding OA to broiler chickens resulted in reduction in feed consumption (Table 2). Total feed consumed were 1295.76, 1241.06, and 1227.03 g for 0, 1, and 2 ppm, respectively, at the end of 4th week. Though there was reduction in feed consumption, it was not statistically significant (*P* < .05).

Similar observations of reduced feed consumption were also made by Hamilton et al. [14], Prior et al. [10], and Hamilton et al. [3].

### 3.3. Feed Conversion

Feed conversion was decreased in OA fed birds (Table 3). However, it was not statistically significant (*P* > .05). Hamilton et al. [3], Kubena et al. [12], and Gibson et al. [13] also reported decreased feed conversion in ochratoxicosis.

### 3.4. Total Protein and Albumin

Feeding OA significantly reduced the total serum protein and albumin levels in broiler chickens (Tables 4 and 5). Similar observations were also made by Manning and Wyatt [7], Huff et al. [4], and Kubena et al. [12].

Huff et al. [4] reported that the total protein and albumin were the sensitive indicator of ochratoxicosis. The mechanism by which OA produced hypoproteinaemia and hypoalbuminaemia is due to inhibition of phenylalanyl transfer-RNA-synthetase with phenylalanine. [15, 16] and renal leakage of albumin resulting from kidney lesions induced by OA [4].

An analysis of the experimental data generated in this investigation clearly point to the adverse effect of OA on feed consumption and growth rate in young broiler chicks. The impairment of protein metabolism would lead to increased susceptibility to various infections and tell upon their production performance. An in-depth study of the immunological alterations evoked by OA in chicks would help to throw more light on the pathogenesis of ochratoxicosis.

## Figures and Tables

**Table 1:** Mean (± SE) weekly body weight (g) of broiler chickens fed ochratoxin.

Ochratoxin (ppm)	Day old (*n* = 20)	Age in days			
		7 (*n* = 20)	14 (*n* = 20)	21 (*n* = 20)	28 (*n* = 20)
0	41.3 ± 0.22	78.0 ± 2.43^a^	190.46 ± 4.05^a^	411.8 ± 4.24^a^	856.3 ± 87.45^a^
1	42.6 ± 0.85	74.6 ± 3.04^a^	175.33 ± 2.45^b^	365.2 ± 3.26^b^	581.0 ± 4.97^b^
2	41.6 ± 1.38	69.4 ± 3.12^b^	151.46 ± 3.60^c^	341.4 ± 3.36^c^	542.4 ± 4.82^c^

Mean values with same superscript do not differ significantly (*P* > .05).

**Table 2:** Mean weekly feed consumption (g) of broiler chickens fed ochratoxin.

Ochratoxin (ppm)	Age in days				Total
	7	14	21	28	
0	53.21	188.92	451.71	601.92	1295.76^a^
1	54.31	183.32	448.62	554.80	1241.06^a^
2	57.36	159.62	432.41	577.64	1227.03^a^

Mean values with the same superscript do not differ significantly (*P* > .05).

**Table 3:** Feed conversion (feed/gain) in broiler chicken fed ochratoxin.

Ochratoxin(ppm)	Age in days				Total
	7	14	21	28	
0	1.44	1.67	2.04	2.46	1.94^a^
1	1.69	1.81	2.36	2.57	2.10^a^
2	2.06	1.94	2.27	2.87	2.28^a^

Mean values with same super script do not differ significantly (*P* > .05).

**Table 4:** Mean (± S.E.) serum total protein g/dL values of broiler chickens fed ochratoxin.

Ochratoxin (ppm)	Weeks				
	1	2	3	4	Mean
0	2.52 ± 0.01	2.39 ± 0.02	2.81 ± 0.01	2.98 ± 0.05	2.71 ± 0.14^a^
1	2.34 ± 0.08	2.2 ± 0.05	2.48 ± 0.01	2.61 ± 0.01	2.41 ± 0.08^b^
2	1.88 ± 0.06	2.32 ± 0.06	2.13 ± 0.03	2.44 ± 0.01	2.19 ± 0.12^b^

Mean values with in columns with the same superscript do not differ significantly (*P* > .05).

**Table 5:** Mean (± S.E.) serum albumin g/dL values of broiler chickens fed ochratoxin.

Ochratoxin (ppm)	Weeks				
	1	2	3	4	Mean
0	1.22 ± 0.01	1.08 ± 0.01	1.29 ± 0.01	1.45 ± 0.05	1.26 ± 0.07^a^
1	1.08 ± 0.01	0.99 ± 0.04	1.22 ± 0.01	1.18 ± 0.04	1.11 ± 0.05^ab^
2	0.99 ± 0.03	1.09 ± 0.03	1.07 ± 0.03	1.05 ± 0.03	1.05 ± 0.02^b^

Mean values with in columns with the same superscript do not differ significantly (*P* > .05).
